# A Novel Knowledge Fusion Ensemble for Diagnostic Differentiation of Pediatric Pneumonia and Acute Bronchitis

**DOI:** 10.3390/diagnostics15172258

**Published:** 2025-09-06

**Authors:** Elif Dabakoğlu, Öyküm Esra Yiğit, Yaşar Topal

**Affiliations:** 1 Research Support and Funding Office, Mugla Sıtkı Koçman University, Mugla 48000, Türkiye; elifdabakoglu@mu.edu.tr; 2 Graduate School of Science and Engineering, Yıldız Technical University, Istanbul 34220, Türkiye; 3 Department of Statistics, Faculty of Arts and Sciences, Yıldız Technical University, Istanbul 34220, Türkiye; 4 Department of Pediatrics, Faculty of Medicine, Mugla Sıtkı Koçman University, Mugla 48000, Türkiye; yasartopal@mu.edu.tr

**Keywords:** pediatric pneumonia, acute bronchitis, ensemble learning, machine learning, meta-learning, stacking ensemble, diagnostic modeling, respiratory infections

## Abstract

**Background:** Differentiating pediatric pneumonia from acute bronchitis remains a persistent clinical challenge due to overlapping symptoms, often leading to diagnostic uncertainty and inappropriate antibiotic use. **Methods:** This study introduces DAPLEX, a structured ensemble learning framework designed to enhance diagnostic accuracy and reliability. A retrospective cohort of 868 pediatric patients was analyzed. DAPLEX was developed in three phases: (i) deployment of diverse base learners from multiple learning paradigms; (ii) multi-criteria evaluation and pruning based on generalization stability to retain a subset of well-generalized and stable learners; and (iii) complementarity-driven knowledge fusion. In the final phase, out-of-fold predicted probabilities from the retained base learners were combined with a consensus-based feature importance profile to construct a hybrid meta-input for a Multilayer Perceptron (MLP) meta-learner. **Results:** DAPLEX achieved a balanced accuracy of 95.3%, an F1-score of ~0.96, and a ROC-AUC of ~0.99 on an independent holdout test. Compared to the range of performance from the weakest to the strongest base learner, DAPLEX improved balanced accuracy by 3.5–5.2%, enhanced the F1-score by 4.4–5.6%, and increased sensitivity by a substantial 8.2–13.6%. Crucially, DAPLEX’s performance remained robust and consistent across all evaluated demographic subgroups, confirming its fairness and potential for broad clinical. **Conclusions:** The DAPLEX framework offers a robust and transparent pipeline for diagnostic decision support. By systematically integrating diverse predictive models and synthesizing both outcome predictions and key feature insights, DAPLEX substantially reduces diagnostic uncertainty in differentiating pediatric pneumonia and acute bronchitis and demonstrates strong potential for clinical application.

## 1. Introduction

Lower respiratory tract infections (LRTIs) remain a leading cause of morbidity and mortality in children worldwide, constituting a significant global health burden. Among these, pneumonia and acute bronchitis pose a persistent diagnostic dilemma, as their overlapping clinical features often hinder accurate differential diagnosis in pediatric settings. This distinction is clinically urgent, given that pneumonia carries substantially higher morbidity and mortality rates. According to the World Health Organization (WHO), pneumonia was responsible for the deaths of 740,180 children in 2019, accounting for approximately 14% of global child mortality [1]. United Nations International Children’s Emergency Fund (UNICEF) data further emphasize this burden, indicating that one child dies from pneumonia every 43 s [2]. In contrast, acute bronchitis is typically less severe, although it frequently mimics key symptoms of pneumonia, including fever, cough, and respiratory distress [3]. This clinical ambiguity not only delays timely intervention but also increases the likelihood of inappropriate antibiotic use, contributing to antimicrobial resistance and placing an avoidable strain on healthcare systems.

The convergence of high LRTI prevalence and diagnostic ambiguity imposes a dual burden, challenging both individual patient management and broader public health systems. While this burden is most pronounced in regions like South Asia and Sub-Saharan Africa [2], the diagnostic challenge is not confined to low-resource settings; it also represents a significant public health concern in emerging economies such as Turkey. In Turkey, LRTIs are a major source of pediatric morbidity. Data from the 2022 Turkey Health Survey reveal that LRTIs were the third most common illness among children aged 0–6 in the preceding six months, with a prevalence of 6.9%, ranking below only upper respiratory tract infections (31.3%) and diarrhea (29.4%) [4]. This substantial prevalence underscores the scale of the diagnostic challenge within the Turkish healthcare system. The frequent co-occurrence of pneumonia and acute bronchitis directly amplifies diagnostic uncertainty, which in turn can lead to suboptimal clinical outcomes and inefficient allocation of healthcare resources. Consequently, there is a pressing need to develop and implement systematic strategies that enhance diagnostic accuracy, not only to improve clinical outcomes in Turkey but also to optimize public health responses in countries with comparable epidemiological profiles.

Enhancing diagnostic accuracy for LRTIs with overlapping symptoms, chiefly pneumonia and acute bronchitis, has become a focal point of clinical and academic research. The limited reliability of physical examination alone—often confounded by shared signs like fever, cough, and tachypnea—necessitates the integration of ancillary data from laboratory and imaging workflows. However, the clinical utility of these data is frequently compromised by challenges in interpretation, stemming from age-dependent physiological variations, clinical heterogeneity, and the inherent limitations of current diagnostic guidelines. This complexity has spurred research aimed at identifying predictor variables with significant discriminative power across clinical, laboratory, and radiological domains. Despite the development of various predictive models using both statistical and machine learning approaches, a critical methodological gap persists. Many existing models are constrained by single-algorithm architectures or limited feature scopes, and they seldom address crucial aspects such as model complementarity or predictive uncertainty, thereby failing to produce robust and generalizable diagnostic tools.

To address the aforementioned diagnostic complexity and methodological gap, this study introduces the Diagnostic Aggregation and Prediction Learning for Explanation (DAPLEX), a novel ensemble-based modeling framework tailored to complex diagnostic differentiation tasks. Its efficacy is demonstrated through application to the clinically critical challenge of distinguishing pediatric pneumonia from acute bronchitis. In contrast to conventional models, which are often constrained by single-algorithm architectures or narrowly defined predictors, DAPLEX employs a multi-phase methodology designed to systematically integrate predictive signals from heterogeneous data domains (clinical, laboratory, and radiological) by leveraging both algorithmic diversity and complementarity through a layered learning architecture. This design aims to enhance diagnostic accuracy, generalizability, and interpretability while offering a robust alternative to traditional approaches.

This study makes three key contributions: (1) it operationalizes algorithmic diversity and complementarity analysis within a clinical diagnostic context; (2) it proposes a transparent and reproducible multi-phase workflow for ensemble construction; and (3) it validates this strategy on a real-world pediatric dataset from Turkey, offering a transferable blueprint for emerging healthcare systems. The remainder of this paper is structured as follows: Section 2 reviews related work, Section 3 details the proposed methodology, Section 4 presents the experimental results, and Section 5 discusses the findings, and Section 6 concludes with key implications and future directions.

## 2. Literature Review

The differentiation of LRTIs, particularly distinguishing pneumonia from acute bronchitis, remains a persistent clinical challenge. This difficulty is exacerbated by the considerable overlap in clinical manifestations and the heterogeneous nature of these conditions in terms of etiology, severity, and therapeutic response. To address this diagnostic ambiguity, the scientific literature has explored a range of methodologies that can be broadly classified into three main domains: (i) traditional diagnostic approaches, which rely on statistical analysis of clinical signs, laboratory biomarkers (e.g., C-reactive protein [CRP], procalcitonin [PCT]), and radiological features; (ii) evidence-synthesis frameworks, including systematic reviews and clinical guidelines, which aim to standardize diagnostic decision-making; and (iii) advanced computational models, particularly machine learning (ML) algorithms, which are designed to integrate high-dimensional data for improved predictive accuracy. While each domain offers valuable insights, a comprehensive review reveals inherent limitations across existing diagnostic paradigms, including restricted predictor integration, insufficient generalizability, and limited attention to model complementarity. These gaps collectively underscore the need for a structured, ensemble-based modeling approach, such as the DAPLEX framework proposed in this study.

The domain of conventional methodologies, fundamentally rooted in standard statistical modeling, has provided a robust foundation for studying pediatric LRTIs. These approaches have effectively highlighted both the complexity of the diagnostic challenge and the areas that warrant further exploration. Epidemiological studies frequently employ tools such as multivariate logistic regression to quantify the public health burden and identify influential risk factors, including socio-environmental determinants and pre-existing medical conditions [5,6,7]. However, translating these population-level insights into precise clinical practice remains difficult due to the etiological complexity of LRTIs, which involve diverse viral and bacterial pathogens that often co-occur, complicating definitive patient-specific diagnoses [8]. In pursuit of greater diagnostic precision, researchers have rigorously evaluated biomarkers such as CRP and PCT using Receiver Operating Characteristic (ROC) curve analyses. These studies consistently demonstrate that the diagnostic utility of these biomarkers varies significantly depending on the clinical context, with typically higher performance observed in distinguishing bacterial pneumonia from acute bronchitis [9]. In addition, ongoing investigations are exploring novel biomarkers tailored to specific clinical scenarios. One example includes the identification of inflammatory mediators in pleural fluid from patients with parapneumonic effusion [10]. Traditional statistical methods have also proven valuable in prognostic assessment. Survival analyses, particularly those using Cox proportional hazards regression, have been employed to identify predictors of adverse outcomes, such as prolonged hospitalization [11]. However, critical management decisions, such as setting universally optimal oxygen saturation thresholds, continue to exhibit variability in clinical consensus [12].

Despite their foundational contributions, conventional statistical methods present critical limitations in the context of pediatric LRTI diagnosis. Their reliance on linear assumptions restricts the capacity to reflect the intricate, non-linear relationships that govern biological variability in disease presentation. Moreover, their tendency to examine clinical, laboratory, and imaging predictors in isolation precludes the discovery of meaningful interactions that emerge only when data from multiple domains are analyzed collectively. These limitations highlight the need for an integrative diagnostic framework that not only accommodates data complexity and heterogeneity but also models the underlying non-linear dynamics more effectively.

The second domain, evidence-synthesis frameworks, serves to unify and critically evaluate the extensive data emerging from individual studies, thereby informing both clinical decision-making and health policy. Key applications include systematic reviews and meta-analyses, which have assessed the diagnostic utility of various methods. These studies have highlighted, for instance, the limited specificity of isolated clinical signs in diagnosing pneumonia [13], and have similarly confirmed that the effectiveness of biomarkers such as CRP and PCT remains moderate when used as standalone indicators [14]. This body of work also encompasses the development of comprehensive clinical guidelines by prominent organizations such as the World Health Organization (WHO), the Pediatric Infectious Diseases Society and the Infectious Diseases Society of America (PIDS/IDSA), and the Turkish Thoracic Society, which aim to standardize care through evidence-based protocols for diagnosis, antibiotic selection, and complication management [15,16]. On a global scale, landmark initiatives such as the Global Burden of Disease (GBD) study have quantified the impact of lower respiratory tract infections (LRTIs) [17], while complementary analyses have highlighted their long-term respiratory consequences [18].

While evidence-synthesis frameworks form a cornerstone of pediatric respiratory care, they also reveal important areas where further refinement may be necessary. One key limitation is that these frameworks are primarily designed to guide decision-making at the population level, which can make it challenging to address the full spectrum of clinical variation seen in individual patients. For example, although rule-based diagnostic criteria are crucial for ensuring standardization, they may not offer the resolution required to navigate atypical or overlapping presentations such as the clinical similarities between viral bronchiolitis and bacterial pneumonia. In addition, meta-analyses often report that even well-established biomarkers demonstrate only moderate diagnostic performance when used in isolation. The continued reliance on empirical treatment further reflects this persistent diagnostic complexity, a challenge also evident in clinical management areas where consensus remains elusive, such as the determination of ideal oxygen saturation thresholds [12]. Collectively, these observations suggest that complementing existing frameworks with data-driven, patient-specific analytical tools may help address current limitations and enhance diagnostic precision.

The final domain, advanced computational models, has emerged to address the limitations of traditional statistical methods by leveraging the power of ML to analyze complex, high-dimensional data. ML applications in pediatric respiratory diagnosis range from interpretable models like decision trees to sophisticated ensemble techniques. For instance, simple yet effective Decision Tree (DT) models have been developed to differentiate pneumonia from acute bronchitis based solely on clinical symptoms, prioritizing practicality in resource-limited settings [19]. Concurrently, more powerful ensemble methods such as Random Forest (RF) and Extreme Gradient Boosting (XGBoost) have demonstrated high predictive accuracy. These models have been used to diagnose pneumonia by integrating extensive biomarker and laboratory data, often employing techniques like feature selection and data balancing [20], as well as identifying key determinant factors from large-scale demographic and health surveys [21]. Beyond binary diagnosis, these advanced algorithms are applied to more nuanced clinical questions. The XGBoost algorithm, for example, has proven effective in predicting specific causative pathogens from routine clinical predictors, with predictions made transparent through explainability methods such as SHAP (Shapley Additive Explanations), which elucidate feature contributions [22]. This interpretable approach has also been successfully applied to the early detection of challenging conditions like refractory Mycoplasma pneumoniae pneumonia [23]. In parallel, RF have excelled at predicting disease severity and the prospective need for Intensive Care Unit (ICU) admission, thereby enabling early risk stratification of patients [24]. This capacity for risk stratification has been further demonstrated in predicting disease severity in patients with COVID-19 pneumonia, where ensemble models also showed high predictive power [25].

While advanced computational studies showcase the significant potential of ML, a closer examination of their methodology reveals a persistent gap. The construction of high-performing ensembles often lacks a systematic and transparent framework. Many approaches either focus on optimizing a single powerful algorithm or combine models without a structured process for ensuring algorithmic diversity across different model families. More critically, the principle of complementarity, which describes the ability of base learners to correct one another’s errors, is rarely quantified or leveraged as a primary criterion for model selection. The predominant focus remains on individual model performance, overlooking the principle that a collection of diverse, complementary models often yields a more robust and generalizable solution. This reveals a crucial limitation in the current literature: the absence of a structured workflow that systematically operationalizes both diversity and complementarity analysis for robust ensemble construction.

In summary, this review reveals a critical gap in the literature: the absence of a structured and transparent framework for ensemble construction in clinical diagnostics. Previous studies have certainly advanced pediatric LRTI diagnostics by integrating biomarkers, imaging, and ML algorithms; however, these contributions remain fragmented. For example, some have focused on probability-level fusion with post hoc explanations (e.g., XGBoost with SHAP [22]), while others emphasized risk stratification with ensemble trees [24,25]. Similarly, simple yet effective Decision Tree (DT) models have been developed to differentiate pneumonia from acute bronchitis based solely on clinical symptoms [19], while more powerful ensembles like RF and XGBoost have demonstrated high predictive accuracy by integrating extensive biomarker and laboratory data [20,21]. However, these studies seldom provide a systematic process for constructing ensembles based on both diversity and complementarity, and almost none integrate probability-level outputs with feature-level insights in a unified architecture. In contrast, DAPLEX explicitly operationalizes this dual perspective by combining prediction-level outputs with a consensus ranking of feature importance profiles, thereby linking performance with interpretability in a single, unified workflow.

To our knowledge, this represents one of the first ensemble frameworks in pediatric respiratory diagnostics that not only quantifies complementarity among learners but also fuses prediction-level and feature-level signals in a transparent and reproducible manner. This positioning is further reinforced when compared to prior works [19,20,21,22,23,24,25], which, despite their valuable contributions, have either remained limited to probability-level fusion, isolated feature interpretation, or task-specific stratification rather than offering a holistic architecture. Building on this gap, the DAPLEX framework operationalizes a multi-phase, dual-layered knowledge fusion strategy that systematically integrates pruning of unstable learners, formal complementarity analysis, and a hybrid predictor–prediction fusion process. By combining prediction-level outputs (the “what”) with a consensus ranking of feature importance profiles via Borda aggregation (the “why”), DAPLEX aligns model performance with interpretability in a unified workflow. In this way, DAPLEX directly improves upon existing diagnostic paradigms by unifying predictive robustness with interpretability, thereby addressing gaps that prior methods left unresolved.

## 3. Materials and Methods

**Figure 1:**
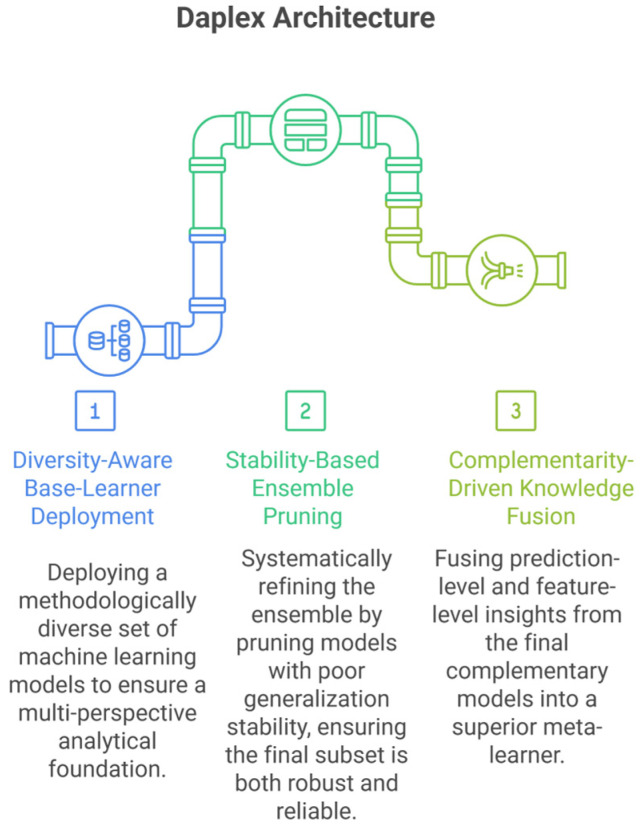
DAPLEX Architecture.

This study introduces DAPLEX, a structured diagnostic modeling framework composed of three sequential phases. Each phase is engineered to progressively refine the model by leveraging distinct machine learning methodologies to extract complementary diagnostic value from clinical data. The core phases of the framework are as follows and are illustrated in Figure 1.

Phase I: Diversity-Aware Base-Learner Deployment: This phase aims to ensure broad representational diversity by deploying base learners from distinct algorithmic families, including bagging-based ensembles, boosting models, kernel-based classifiers, instance-based methods, and probabilistic models. By leveraging fundamentally different learning paradigms, the framework captures heterogeneous diagnostic patterns that may be missed by more homogeneous ensembles.

Phase II: Stability-Based Ensemble Pruning: A rigorous pruning procedure, based on 5-fold cross-validation results from the training data, was employed to retain only those learners exhibiting both high performance on the validation folds and robust generalization stability, quantified as a minimal gap between training and validation scores.

Phase III: Complementarity-Driven Knowledge Fusion: In the final phase, a meta-model is constructed by integrating probabilistic predictions and feature-level insights from the pruned ensemble. Model complementarity is formally quantified to guide this integration, resulting in a final decision that is both robust and clinically meaningful.

The following subsections detail the study cohort, data preprocessing, technical implementation of each framework phase, and model evaluation procedures.

### 3.1. Study Design and Ethical Considerations

This study was designed as a retrospective, single-center cohort analysis. The research protocol was reviewed and approved by the Institutional Review Board of Muğla Sıtkı Koçman University (Approval No: 230103; Date: 21 December 2023). All procedures were conducted in accordance with the ethical standards of the Declaration of Helsinki. To ensure patient confidentiality, all records were anonymized prior to data processing and analysis.

### 3.2. Data Acquisition and Patient Cohort

Patient data were retrospectively extracted from the electronic health records (EHR) of Muğla Sıtkı Koçman University Training and Research Hospital, a tertiary care center in Turkey. The dataset spans the period from January 2019 to December 2023. A total of 1000 pediatric admissions with lower respiratory tract infection (LRTI)-related diagnoses were initially screened. After applying predefined eligibility criteria, 868 cases were included in the final analytic cohort. Inclusion required a definitive diagnosis of either pneumonia or acute bronchitis, as documented by a pediatrician. Cases were excluded if essential predictor variables were missing, such as laboratory results, physical examination findings, or radiological data. Patients with comorbid respiratory conditions that could interfere with diagnostic differentiation, including asthma, cystic fibrosis, or tuberculosis, were also excluded. All data were obtained during routine clinical care and reflect standard diagnostic practices in pediatric respiratory medicine.

### 3.3. Study Variables and Baseline Analysis

For each patient in the final cohort, a set of 28 predictor variables was systematically extracted from the EHR. These variables were selected based on their clinical relevance to pediatric LRTIs and their consistent availability in routine diagnostic workflows and were categorized into five domains: (i) demographic characteristics, (ii) presenting symptoms, (iii) physical examination findings, (iv) laboratory parameters, and (v) radiological assessments. The primary outcome for the study was the final clinical diagnosis, operationalized as a binary classification task: pneumonia (coded as 1) versus acute bronchitis (coded as 0). The final analytic cohort consisted of 474 (54.6%) pneumonia cases and 394 (45.4%) acute bronchitis cases.

A comprehensive summary of the baseline characteristics for all variables is provided in the Supplementary Materials (Table S1). To identify significant differences between the pneumonia and bronchitis groups, baseline characteristics were compared using Welch’s *t*-test for continuous variables and Pearson’s Chi-square test for categorical variables. Welch’s *t*-test was chosen for its robustness to unequal variances and sample sizes. Continuous variables were reported as mean ± standard deviation, while categorical variables were presented as frequency (*n*) and percentage (%).

### 3.4. Data Preprocessing and Splitting

Prior to model development, the dataset was first split into a training set (80%) and an independent holdout test set (20%) with stratification by final diagnosis to preserve the original class distribution (54.6% pneumonia, 45.4% acute bronchitis) across subsets. A single fixed partition was used in all subsequent modeling and evaluation steps to ensure reproducibility and to isolate performance differences attributable solely to model characteristics.

Following the data split, a standardized preprocessing pipeline was developed. The final analytic cohort contained no missing values for the selected predictors; therefore, the pipeline focused on feature transformation. To prevent any data leakage from the test set, the parameters for all transformations (e.g., means and standard deviations for scaling) were learned exclusively from the training data. This fitted pipeline was then applied to transform both the training and the test sets identically. The pipeline implemented two primary transformations. For all nominal categorical features, one-hot encoding was applied to generate binary indicator variables, mitigating the risk of imposing artificial ordinal relationships In parallel, z-score standardization was used to normalize continuous predictors, rescaling them to a mean of zero and a standard deviation of one. This procedure ensured that all features contributed comparably to model training, regardless of their original scales.

### 3.5. DAPLEX Architecture and Analytical Framework

The DAPLEX framework conceptualizes the diagnostic task as a structured, three-phase pipeline. The process begins with the deployment of diverse base learners drawn from distinct ML families to capture heterogeneous diagnostic signals. These learners are then subjected to rigorous evaluation procedures to assess their predictive accuracy, generalizability, and mutual complementarity. In the final phase, a meta-learner integrates the ensemble’s collective outputs through a structured fusion mechanism that combines probabilistic predictions and feature-level insights into a unified meta-representation. The technical implementation of each phase is detailed in the following subsections. The entire analytical workflow was implemented in Python (version 3.12.3; Python Software Foundation, Wilmington, DE, USA) using the scikit-learn library (version 1.6.1), the pandas library (version 2.2.2), the XGBoost library (version 2.1.4), the NumPy library (version 1.26.4), the SciPy library (version 1.13.1), the Matplotlib library (version 3.9.2), and the Seaborn library (version 0.13.2).

#### 3.5.1. Phase I: Diversity-Aware Base-Learner Deployment

Phase I of the DAPLEX framework is grounded in the principle of diversity-aware ensemble design. This strategy, widely acknowledged for its ability to enhance generalization, promotes algorithmic heterogeneity to capture complementary diagnostic patterns that may be missed by homogeneous models [26,27,28]. Accordingly, six base classifiers were selected to represent distinct learning paradigms, including tree-based ensembles (bagging and boosting), margin-based geometric models, instance-based learners, and probabilistic classifiers. The rationale for each selection was based on a combination of established theoretical diversity and empirical evidence of strong performance in similar clinical prediction tasks.

The core of the ensemble is formed by two powerful tree-based models representing distinct strategies: bagging and boosting. The first, Random Forest (RF), employs bootstrap aggregation to generate a large number of decorrelated DTs, a technique known to enhance robustness and generalization [29,30]. RF was selected due to its consistent top-tier performance in diverse clinical applications, where it often outperforms other classifiers [21,31,32,33,34,35,36,37]. This is complemented by Extreme Gradient Boosting (XGBoost), a sequential boosting model that iteratively improves performance by correcting residual errors. XGBoost was included for its state-of-the-art accuracy and regularization capabilities, as supported by a broad range of clinical prediction studies [23,25,38,39,40,41,42]. To extend algorithmic diversity beyond tree-based methods, the ensemble was further augmented with classifiers representing three additional paradigms. First, a margin-based geometric approach was incorporated via two support vector machine (SVM) variants—Radial Basis Function (RBF) and Polynomial kernels (SimplePoly)—each designed to identify an optimal separating hyperplane in a transformed feature space. SVMs have demonstrated strong performance across a range of clinical prediction tasks [43,44,45,46,47]. Second, the instance-based learner K-Nearest Neighbors (KNN) was employed, which classifies samples based on the majority vote of their nearest neighbors in the feature space. KNN’s simplicity and interpretability make it a useful component in hybrid diagnostic systems, particularly when used with feature selection strategies [48,49]. Lastly, Gaussian Naive Bayes (GNB) was included to provide a probabilistic perspective. Despite its assumption of feature independence, GNB is valued for its computational efficiency and has been shown in several systematic reviews to perform comparably with more complex classifiers in medical applications [50,51,52,53,54]. In summary, each classifier was chosen to contribute a unique algorithmic perspective, ensuring the initial pool was both diverse and populated with models empirically validated in clinical contexts.

**Figure 2:**
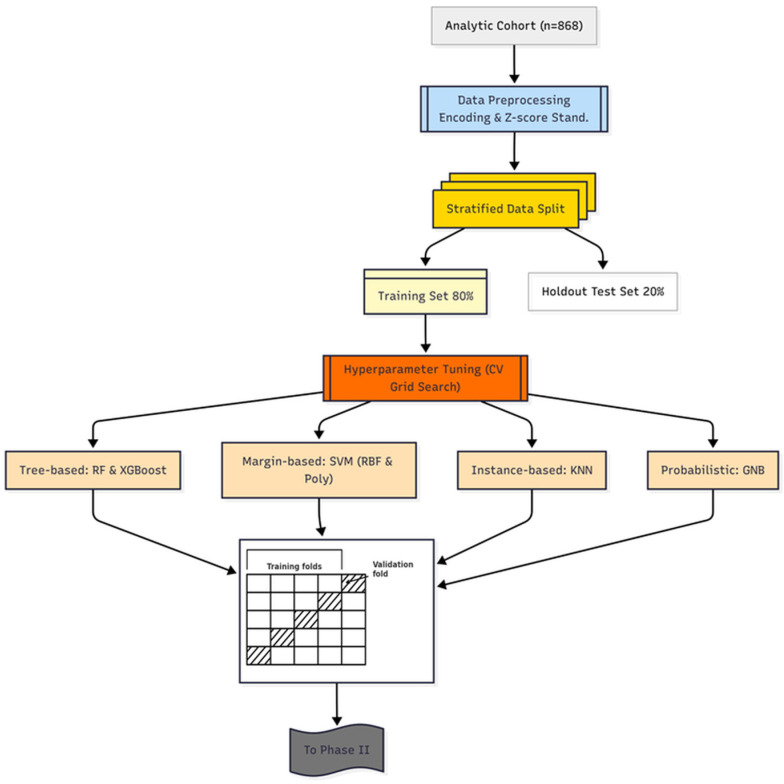
DAPLEX-Phase I Workflow.

To ensure optimal performance, the hyperparameters for each base learner were systematically tuned using a 5-fold cross-validated grid search (GS) strategy (GridSearchCV), performed exclusively on the training dataset. This k-fold scheme was deliberately chosen as it provides a robust estimate of model performance while maintaining a favorable balance between bias, variance, and computational cost for a dataset of 868 patients. Alternative schemes, such as leave-one-out or 10-fold cross-validation, would either yield fewer stable estimates or impose unnecessary computational burden in this setting. Balanced accuracy (BA) was chosen as the optimization metric to mitigate predictive bias toward the more prevalent pneumonia class. The optimal hyperparameter set identified for each model was then used to train the final base learner on the entire training set before proceeding to the next phase. A schematic overview of the Phase I workflow is presented in Figure 2, illustrating the systematic deployment of diverse base learners to capture complementary diagnostic patterns. This diversity serves as a strategic foundation for effective pruning in Phase II and integrative fusion in Phase III.

#### 3.5.2. Phase II: Stability-Based Ensemble Pruning

The second phase of the DAPLEX framework is dedicated to stability-based ensemble pruning, a critical step intended to refine the initial pool of optimized base learners from Phase I into a parsimonious yet high-performing subset [55]. To ensure a robust selection process free from data leakage from the holdout test set, this evaluation was performed exclusively on the results from the 5-fold cross-validation conducted on the training data. Accordingly, each optimized model was systematically assessed by evaluating the generalization stability of key metrics representing two core dimensions of model behavior: discriminative performance and calibration reliability.

The first core dimension, discriminative performance, refers to a model’s ability to accurately distinguish between pneumonia and acute bronchitis. To quantify this, the average performance of each optimized model was evaluated across the validation folds of the 5-fold cross-validation using several complementary metrics. These included Balanced Accuracy (BA), the F1-score, and the area under the receiver operating characteristic curve (ROC-AUC). Sensitivity (also known as the true positive rate, TPR) represents the proportion of actual pneumonia cases correctly identified by the model, whereas specificity (true negative rate, TNR) reflects the proportion of acute bronchitis cases accurately excluded. BA, calculated as the average of sensitivity and specificity, was adopted as the primary performance metric due to its robustness in the presence of class imbalance. Additionally, the F1-score, defined as the harmonic mean of precision and recall [56], summarizes a model’s ability to balance false positives and false negatives. Here, precision refers to the proportion of predicted pneumonia cases that were truly pneumonia, while recall corresponds to sensitivity. Finally, ROC-AUC was included as a threshold-independent metric that captures model performance across varying decision thresholds by plotting sensitivity against 1—specificity; the AUC value summarizes the overall discriminative capacity of the model [57]. The equations used to compute these metrics are listed below, where TP, TN, FP, and FN represent the counts of true positives, true negatives, false positives, and false negatives, respectively.

$$\mathrm{Sensitivity}\left(\mathrm{Recall}\right)=\frac{\mathrm{TP}}{\mathrm{TP}+\mathrm{FN}}$$

$$\mathrm{Specificity}=\frac{\mathrm{TN}}{\mathrm{TN}+\mathrm{FP}}$$

$$\mathrm{Precision}=\frac{\mathrm{TP}}{\mathrm{TP}+\mathrm{FP}}$$

$$\mathrm{Balanced}\;\mathrm{Accuracy}\;(\mathrm{BA})=\frac{\mathrm{Sensitivity}+\mathrm{Specificity}}{2}$$

$$F1\text{-}\mathrm{Score}=2\;\times \;\frac{\mathrm{Precision}\;\times \;\mathrm{Recall}}{\mathrm{Precision}+\mathrm{Recall}}$$

$$\mathrm{ROC}\text{-}\mathrm{AUC}=\int_{0}^{1}\mathrm{TPR}\left(\mathrm{FPR}\right)d(\mathrm{FPR})$$

The second core dimension, calibration reliability, is defined as the degree to which a model’s predicted probabilities reflect the actual likelihood of outcomes. This was assessed across the validation folds using the Brier score, a proper scoring rule that quantifies the mean squared difference between predicted probabilities (p_i_) and observed binary outcomes (o_i_) across all N patients. Lower Brier scores indicate better-calibrated models [58,59], and the score is calculated using the following formula:

$$\mathrm{Brier}\;\mathrm{Score}\;=\;\frac{1}{N}\sum_{i\;=\;1}^{N}{\left(p_{i}\;-\;o_{i}\right)}^{2}$$

Finally, generalization stability was assessed to evaluate each model’s resilience to overfitting and its capacity to generalize. This was quantified by comparing a model’s score on the training folds (Mean Score_train_) with its score on the corresponding validation folds (Mean Score_validation_) during the 5-fold cross-validation. This stability gap analysis was conducted for all key discriminative performance and calibration metrics to obtain a holistic view of each model’s reliability. The stability gap for each key metric (${∆}_{\mathrm{metric}}$) was calculated as follows:

$${∆}_{\mathrm{metric}}\;=\;\left|{Mean\;Score}_{train,\;CV}-{Mean\;Score}_{val,\;CV}\right|$$

Based on this comprehensive evaluation, base learners exhibiting suboptimal characteristics were excluded from further integration. Models were pruned if they were flagged for weak generalization stability, a condition met if the stability gap for a given metric surpassed a predefined threshold. To ensure fairness, this stability check was systematically performed across all computed metrics. However, the definitive pruning decision was anchored to BA, with an exclusion threshold of Δ_BA_ > 0.05. The rationale for this choice lies in BA’s unique capacity to jointly account for sensitivity and specificity under potential class imbalance, which is critical in the present diagnostic context. Importantly, other key metrics such as ROC-AUC and F1-score consistently reinforced the same relative ranking of models, thereby validating the use of BA as a robust and representative criterion for pruning.

**Figure 3:**
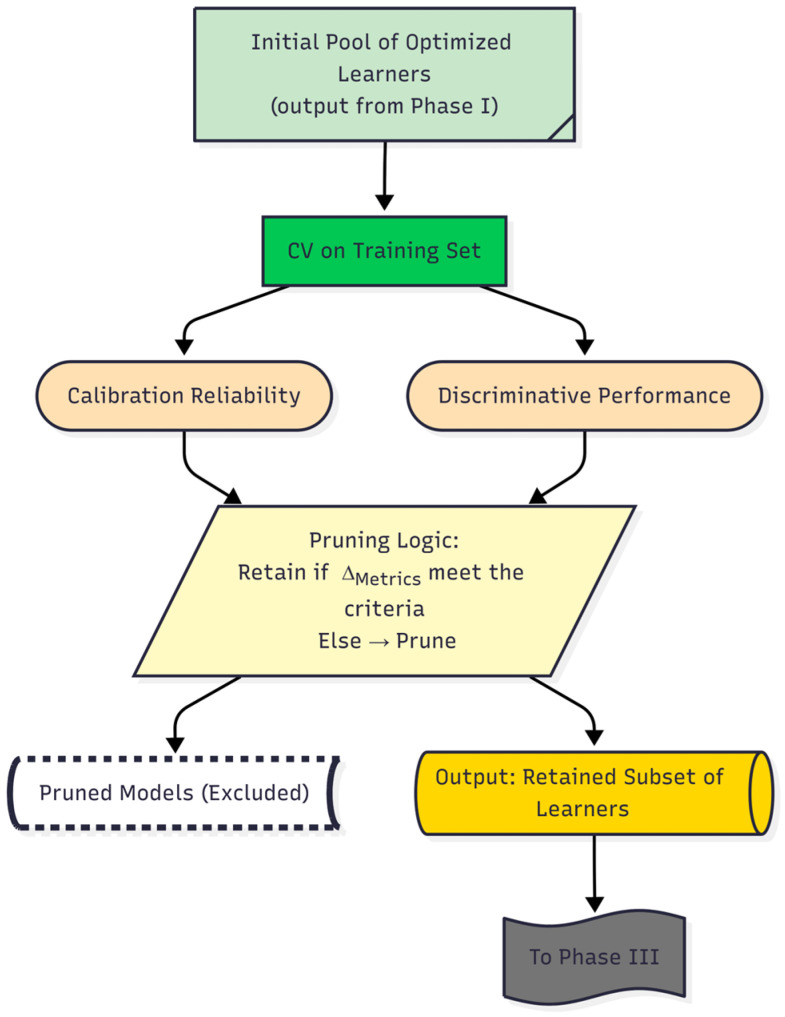
Daplex-Phase II Workflow.

Through this step, the remaining subset of models that satisfied the stability criterion was retained for subsequent integration. These selected models form the core ensemble candidates for Phase III. A schematic overview of the Phase II workflow is presented in Figure 3, which outlines the critical pruning mechanism that establishes a high-confidence foundation for the final stage of the DAPLEX framework—complementarity-driven knowledge fusion.

#### 3.5.3. Phase III: Complementarity-Driven Knowledge Fusion

The final phase of the DAPLEX framework transitions from model selection to knowledge fusion, a process designed to leverage the complementary strengths of the pruned base learners and synthesize a more accurate and robust final decision system. This phase consists of two primary stages: first, a formal complementarity analysis to verify that the retained learners offer diverse predictive insights, and second, a knowledge fusion process designed to synthesize not only what the models predict (their probabilistic outputs) but also the rationale behind those predictions (feature-level insights) into a final, unified diagnostic model.

To formally assess model complementarity, both statistical and information-theoretic measures were employed. The Friedman test was utilized to determine if statistically significant differences existed among the models’ cross-validation performance rankings, indicating non-redundancy. In parallel, Jensen-Shannon Divergence (JSD) was calculated to quantify the dissimilarity between the full probabilistic outputs of model pairs [60]. These analyses provided quantitative evidence that the ensemble members were sufficiently distinct, justifying their subsequent integration.

Insights from the complementarity analysis informed the core integration mechanism: a hybrid knowledge fusion strategy that extends conventional stacking. This approach generates an enriched meta-input by synthesizing two distinct sources of information: (i) prediction-level meta-features and (ii) feature-level meta-features. This combination enables the meta-learner not only to learn from the predictive behavior of the base models but also to incorporate the underlying rationale behind their decisions, significantly enriching the information available for the final classification.

The first of these sources, the prediction-level meta-features, consisted of the out-of-fold (OOF) predicted probabilities from each base learner. This technique is standard in stacked generalization to mitigate information leakage to the meta-learner, thereby reducing overfitting risk—a principle established by the Super Learner algorithm [61,62]. The second source, a consensus-based feature importance profile, was derived using a multi-step process. First, feature rankings for each base model were generated via permutation importance, a model-agnostic technique valued for its unbiased estimates [63,64]. These individual rankings were then aggregated into a unified hierarchy using the Borda count method, a consensus-based voting system chosen for its robustness in combining disparate preference lists. This approach is particularly well-suited for this context, as it creates a stable, aggregate feature importance profile by giving equal weight to the unique predictive logic of each heterogeneous learner, thereby mitigating the biases of any single model.

A wide variety of algorithms have been employed as meta-learners in stacking ensembles, ranging from single classifiers such as Decision Tree, Naïve Bayes, and SVM [65,66,67,68,69,70], to more complex ensemble-based systems including RF, XGBoost, and LightGBM [71,72]. Multi-Layer Perceptrons (MLPs) are also frequently adopted as meta-learners [73,74,75] and have been reported to outperform alternative choices in clinical prediction contexts [76,77]. This diversity in the literature indicates that there is no single “golden standard” meta-learner, and the most appropriate choice depends on the data structure and problem setting [78]. Within the DAPLEX framework, the MLP was deliberately selected as the meta-learner due to its distinct advantages in addressing the specific challenges of the application. First, the complex, non-linear relationships inherent in clinical diagnostic data often constrain the predictive capacity of simpler, linear models. An MLP, with its inherent ability to capture such intricate patterns, was therefore regarded as a more suitable candidate. Second, the MLP architecture is particularly well-suited to process the unique, hybrid meta-input of the framework, which combines probabilistic outputs from base learners with raw, high-importance feature values. This capacity to effectively integrate and uncover complex relationships within heterogeneous sources of information establishes the MLP as a technically robust choice for the complementarity-driven knowledge fusion central to DAPLEX.

**Figure 4:**
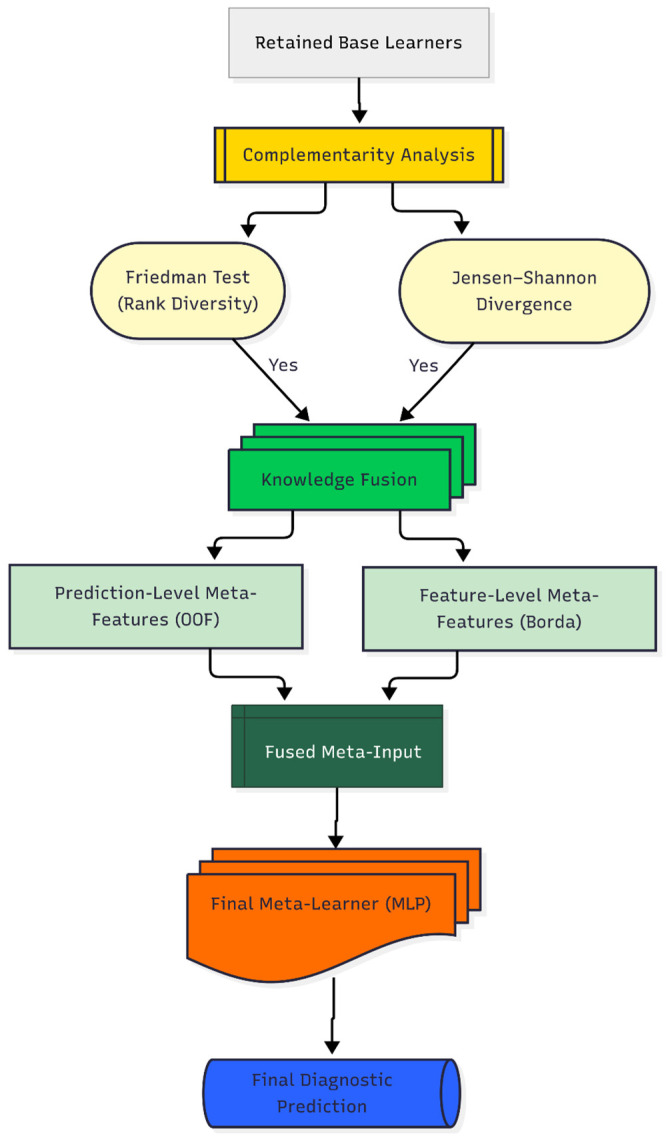
Daplex-Phase III Workflow.

By incorporating both what the base models predicted (their probabilistic outputs) and why certain predictors were deemed important (the aggregated feature importance profile), the MLP served as the integrative layer that transformed the ensemble’s collective knowledge into a unified and high-confidence diagnostic decision. The MLP was trained following a rigorous optimization protocol: the fused meta-input was first standardized using z-score normalization, and its hyperparameters were subsequently tuned via an exhaustive grid search with 5-fold cross-validation, guided by optimizing for BA. The final output of this trained MLP constitutes the DAPLEX framework’s diagnostic prediction. A schematic of this complementarity-driven knowledge fusion process is presented in Figure 4, illustrating the complete pipeline designed to synthesize the ensemble’s collective intelligence into a single, high-confidence diagnostic prediction.

Moreover, to ensure that this complementarity-driven fusion remains adaptable across different clinical datasets, the DAPLEX framework was deliberately designed with modular flexibility. At the level of data acquisition, it is data-agnostic and accommodates heterogeneous diagnostic domains (demographic, symptom history, physical examination, laboratory, radiological), with the option to incorporate additional predictors without altering the workflow. Preprocessing and splitting are handled through a transparent pipeline using stratified partitioning and a scikit-learn ColumnTransformer, where transformations (e.g., scaling, encoding, imputation) can be modified as needed to match dataset characteristics. Finally, hyperparameter search spaces are defined in centralized configuration dictionaries, allowing researchers to easily adjust optimization strategies and metrics. In addition, the framework’s modularity extends to Phase III, where complementarity metrics (e.g., Kullback-Leibler Divergence, correlation matrices), feature-importance methods (e.g., SHAP), and the hybrid fusion architecture (e.g., expanding the dual-stream input) are interchangeable, enabling adaptation to evolving methodological preferences. This modular design ensures that DAPLEX can be applied to datasets of varying size, balance, and feature composition while maintaining methodological transparency and reproducibility.

## 4. Results

The DAPLEX framework yielded high diagnostic accuracy in differentiating pediatric pneumonia from acute bronchitis. This section reports the empirical findings supporting this outcome, beginning with a summary of the patient cohort’s baseline characteristics, which revealed statistically significant differences between diagnostic groups. Subsequent subsections detail the analytical results of each phase, including base learner performance, the complementarity analysis of the pruned ensemble, and the final diagnostic performance of the fully integrated DAPLEX model.

### 4.1. Baseline Characteristics of the Study Cohort

The baseline demographic and clinical characteristics of the 868 patients included in the study are presented in Supplementary Materials (Table S1). Univariate comparisons revealed numerous statistically significant differences between the pneumonia (*n* = 474) and acute bronchitis (*n* = 394) cohorts. Patients diagnosed with pneumonia were, on average, significantly older (mean: 53.2 ± 2.4 months) than those with acute bronchitis (35.9 ± 2.0 months, *p* < 0.001) and had markedly elevated CRP levels (40.4 ± 3.1 mg/L vs. 14.2 ± 1.1 mg/L, *p* < 0.001). Fever was also significantly more prevalent in the pneumonia group (77.2% vs. 38.1%, *p* < 0.001), as was myalgia (22.4% vs. 16.5%, *p* = 0.038).

Distinctive physical examination findings further differentiated the two groups. Auscultatory findings provided a striking contrast: crackles were a hallmark of pneumonia (91.4% vs. 17.8%), while rhonchus (18.6% vs. 98.7%) and prolonged expiration (14.8% vs. 96.7%) were almost exclusively observed in acute bronchitis patients (all *p* < 0.001). Signs of increased work of breathing—including respiratory distress, tachypnea, tachycardia, nasal flaring, and intercostal retractions—were more frequently observed in the acute bronchitis group (all *p* < 0.001). Hyperpnea was also more common among acute bronchitis patients (85.8% vs. 65.2%, *p* < 0.001), although its diagnostic specificity remains limited.

Radiological assessments further reinforced the diagnostic distinction. Pneumonic infiltration was characteristically observed in pneumonia cases (85.7% vs. 0.8%), whereas increased aeration and air bronchogram were predominant in the acute bronchitis cohort (12.7% vs. 92.1%) (*p* < 0.001).

Collectively, these findings confirm the existence of rich, multi-domain diagnostic patterns distinguishing the two cohorts, providing a robust foundation for the primary analysis of this study. The subsequent analyses evaluate whether a multivariate modeling approach can effectively harness these complex patterns for robust diagnostic differentiation.

### 4.2. Results of the DAPLEX Modeling Pipeline

The empirical results of the DAPLEX framework are presented sequentially, in alignment with the model’s three-phase architecture. The analysis begins with the performance evaluation of the initial base learners (Phase I), followed by the results of the ensemble pruning and complementarity analysis (Phase II & III), and culminates in the final diagnostic performance of the fully integrated model.

Before presenting the empirical results, it is important to confirm that the training and independent hold-out test sets were comparable in their baseline characteristics to address the potential for optimistic bias associated with the modest sample size. To this end, all predictors were statistically compared between the two subsets using appropriate tests (*t*-test for continuous variables and chi-square test for categorical variables). As summarized in Supplementary Table S2, none of the predictor variables exhibited statistically significant differences (all *p*-values > 0.05), indicating balanced distributions across all domains. This foundational check verifies that the holdout test set is a representative sample of the overall study population, providing confidence that the subsequent performance results are robust and not influenced by sampling bias.

#### 4.2.1. Results of Phase I: Base Learner Performance

In Phase I, the six individually optimized base learners were evaluated to establish their baseline performance and stability, exclusively using a 5-fold cross-validation procedure on the training data. The detailed results of this analysis, presented in Table 1, reveal a diverse landscape of model strengths.

RF emerged as the strongest all-around performer, achieving the highest mean BA (0.948 ± 0.016) and the best precision-recall balance (F1-score: 0.943 ± 0.017). Crucially, it shared the highest specificity (0.991 ± 0.011) and precision (0.990 ± 0.013) with SVM (RBF), indicating an exceptional ability to avoid false positives and provide reliable positive predictions. Furthermore, RF and XGBoost achieved the highest discriminative capacity (ROC-AUC = 0.985), with SVM (RBF) also demonstrating excellent performance. However, the analysis also highlighted a notable heterogeneity in model strengths. For instance, while KNN showed competitive BA, it was identified as a less reliable candidate due to its comparatively weaker discriminative capacity, as evidenced by a lower ROC-AUC relative to RF and SVM (RBF). In contrast, XGBoost distinguished itself by demonstrating the highest stability, consistently achieving the lowest variance across most metrics (e.g., BA SD = ±0.012). Finally, Brier scores indicated that RF provided the most reliable probability estimates (0.056 ± 0.017), confirming its superior calibration compared to other models.

This heterogeneity in model strengths underscored that different learners captured different aspects of the data, providing a strong rationale for the complementarity-driven ensemble approach in Phase III. At the same time, it provided the necessary empirical evidence for the pruning decisions in the subsequent phase, which prioritized models offering a robust balance of high performance and proven stability. The complete hyperparameter search spaces alongside the final selected configurations for each model are provided in the Supplementary Materials (Table S3).

#### 4.2.2. Results of Phase II: Stability-Based Ensemble Pruning

Following the comprehensive evaluation in Phase I, the six base learners were subjected to a quantitative pruning process based on their generalization stability. This step is crucial for ensuring a robust foundation for the final ensemble. The definitive pruning criterion was the stability gap (Δ_BA_), calculated as the difference between the mean balanced accuracy on the training and validation folds during cross-validation. A predefined exclusion threshold of Δ_BA_ > 0.05 was applied to remove models with a significant tendency to overfit.

The results of this analysis are presented in Table 2. While most models demonstrated robust generalization, KNN was flagged as a significant outlier, with a performance drop (Δ_BA_ = 0.0645) that surpassed the stability threshold. Importantly, similar gaps were also observed across other key metrics (F1, Sensitivity, Specificity, Precision, ROC-AUC, and Brier), as illustrated in Supplementary Figure S1, thereby corroborating the pruning decision beyond BA alone. A comprehensive overview of the stability gaps for all evaluated metrics is provided in Supplementary Table S4.

**Figure 5:**
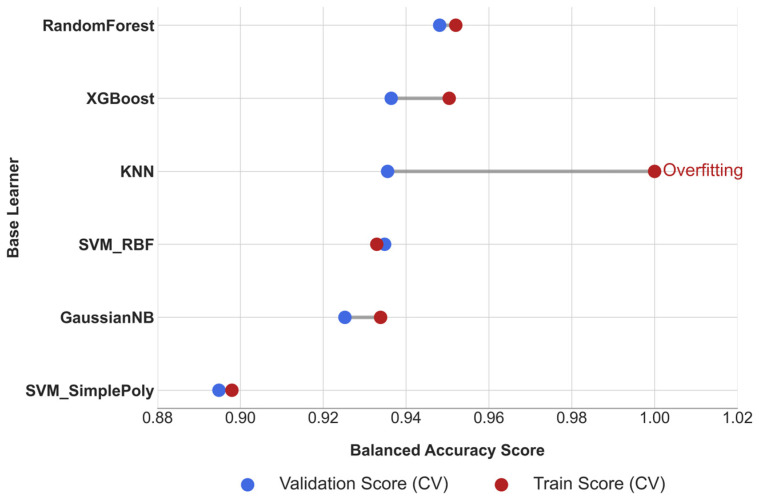
Cross-Validation Stability Analysis of Base Learners.

To complement the numerical results, the dumbbell plot in Figure 5 illustrates the Δ_BA_, with larger gaps indicating a higher risk of overfitting. The plot clearly identifies KNN as an outlier, with a stability gap exceeding the predefined threshold (Δ_BA_ = 0.0645). Consistent patterns across other metrics are provided in Supplementary Figure S1, further supporting this finding.

Consequently, to maintain the integrity and robustness of the final ensemble, the KNN model was pruned from the candidate pool. The remaining five models—RF, XGBoost, SVM-RBF, SVM-Poly, and GNB—constituted the pruned ensemble and were retained for further integration and complementarity analysis in Phase III.

#### 4.2.3. Results of Phase III: Complementarity-Driven Knowledge Fusion

##### Ensemble Complementarity Analysis

**Figure 6:**
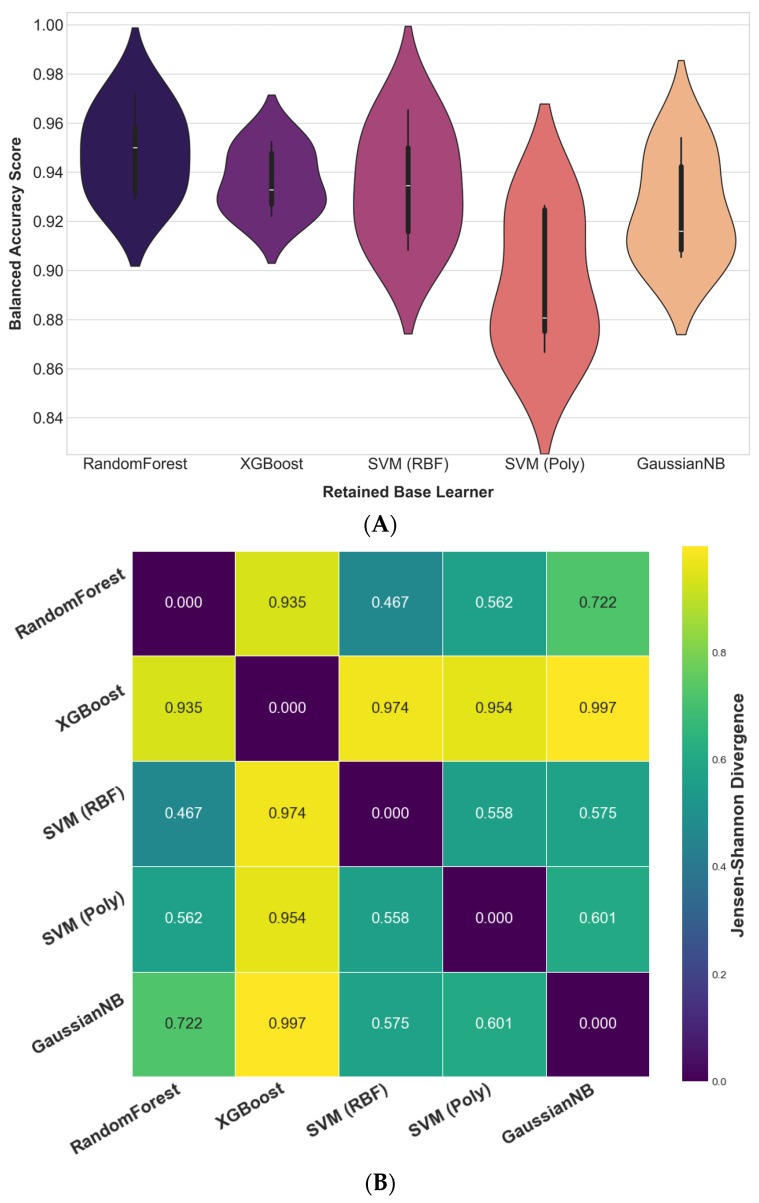
Complementarity assessment of the retained base learners. (**A**) Distribution of 5-fold cross-validation BA scores, used to assess the diversity in the models’ learning behaviors via the Friedman test. (**B**) Pairwise JSD between model outputs, quantifying probabilistic diversity.

The predictive complementarity and diversity of the five retained base learners was formally assessed to justify their integration into the final ensemble. First, a Friedman test on the models’ cross-validation BA scores revealed a statistically significant difference in their performance rankings (χ^2^(4) = 11.80, *p* = 0.019), rejecting the null hypothesis that all models perform equally. This indicates that the learners exhibit distinct learning behaviors. Figure 6A illustrates the distribution of these cross-validation scores, revealing noticeable differences among the models. Both RF and SVM (RBF) achieved high median BA, with RF exhibiting lower variance, reflecting more consistent performance across folds. In contrast, SVM (Poly) demonstrated wider variability and a lower median accuracy; however, this heterogeneity indicates that it captured distinct predictive patterns. Rather than being a limitation, such variability contributed to the diversity of learning signals within the ensemble, enhancing the complementarity essential for robust knowledge fusion.

Second, to assess probabilistic complementarity, pairwise JSD values were computed based on the full predicted probability distributions of each learner. As visualized in the heatmap (Figure 6B), JSD values ranged from 0.467 to 0.997, indicating substantial divergence in the models’ probabilistic reasoning. The consistently high divergence scores (especially between XGBoost and the remaining learners) suggest that the retained models captured markedly different aspects of the data. The absence of near-zero divergence values further indicates that the learners relied on distinct predictive logic, reinforcing their potential for complementarity when integrated.

Collectively, these findings offer robust, multi-perspective evidence that the final ensemble is composed of high-performing yet complementary learners. With model complementarity empirically validated, the analysis now proceeds to evaluate the diagnostic performance of the fully integrated DAPLEX framework.

##### Knowledge Fusion and Feature Importance

Building upon the retained ensemble of five diverse and stable base learners, this phase of the DAPLEX framework leverages their complementarity through a structured knowledge fusion strategy. The fusion process synthesizes two core components: (i) the predictive probabilities from each learner (what the model predicts) and (ii) a consensus-driven ranking of feature importances (why the prediction is made).

**Figure 7:**
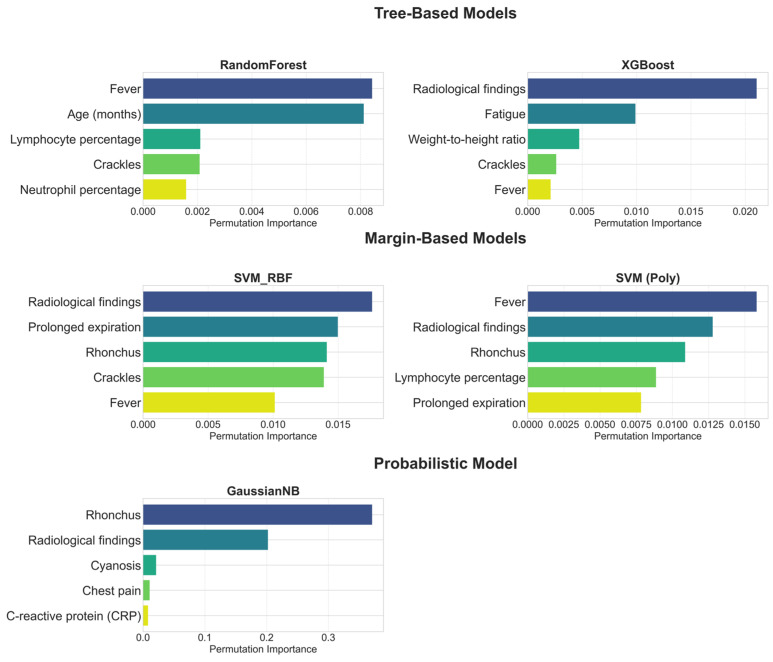
Top 5 Features by Permutation Importance for Each Base Learner.

The feature importance profiles of the individual base learners, presented in Figure 7, offer clinically meaningful insights into their underlying logic. A detailed examination of these profiles demonstrates that the models’ feature hierarchies are not arbitrary but instead closely align with established principles for differentiating pneumonia from acute bronchitis. This alignment is evident across key clinical domains, which are discussed sequentially below.

Radiological Assessment: As shown in Figure 7, radiological findings not only have the highest importance in models such as XGBoost and SVM (RBF), but are also among the top discriminators in nearly all base models. Even in GNB, where rhonchi ranked first, radiological findings still appeared as the second most influential, underlining their robustness across model families. This pattern is fully consistent with clinical expectations, since the presence of infiltration or consolidation on a chest radiograph is recognized as a primary diagnostic criterion for pneumonia [79,80]. In contrast, acute bronchitis is characterized by the absence of consolidation, with chest radiographs often appearing normal or demonstrating only non-specific findings such as peribronchial thickening [81]. This clear distinction explains why Figure 7 depicts radiological features as the most objective differentiators between the two diseases. Therefore, the models’ prioritization of radiological findings is not merely an algorithmic artifact but rather demonstrates a strong alignment with established diagnostic criteria in pediatric clinical practice [79,80].

Physical Examination: As illustrated in Figure 7, base models also captured the discriminative value of physical examination findings. Crackles, which reflect fluid accumulation in the alveoli, were consistently ranked among the top five predictors in multiple models, in line with their recognition as a characteristic sign of pneumonia in pediatric clinical guidelines [15]. By contrast, rhonchi, which were ranked highest particularly in the GNB model, reflect their association with mucus in the larger airways, a hallmark feature of acute bronchitis [81]. The ability of the models to use both signals discriminatively highlights their capacity to comprehend the complex clinical picture more comprehensively. Furthermore, the inclusion of findings such as prolonged expiration in margin-based models and cyanosis in the GNB model indicates that the models are also sensitive to more severe clinical signs like changes in respiratory patterns and hypoxia. Thus, physical examination findings provided a distinct discriminative contribution, demonstrating sensitivity that extends beyond objective radiological findings to encompass clinical examination findings.

Symptomatology: Symptomatological signs also play an important role in diagnostic differentiation, particularly the fever feature. Indeed, this feature was ranked first as the most important discriminator in models such as RF and SVM (Poly) (Figure 7). However, the more variable ranking of this feature in other models reflects its dual role in clinical practice: while fever is an indispensable signal as a common, core symptom in both diagnoses, its standalone discriminative power is not as definitive as that of radiological findings or specific physical examination signs [82]. Chest pain, as detected by GNB (Figure 7), reveals its importance as a more specific symptom that can indicate pneumonia complications such as pleurisy [15].

Laboratory Parameters: Laboratory findings also emerged as significant discriminators for specific models. Notably, the RF and SVM (Poly) models included lymphocyte percentage and neutrophil percentage among their top five variables (Figure 7), which can indicate the etiology of the infection (bacterial/viral) [9]. CRP, an inflammatory marker, was found to be significant by the GNB model, consistent with evidence supporting its utility in differentiating bacterial from non-bacterial lower respiratory tract infections [9]. This highlights how the models constituting the ensemble, each with its unique approach, use hematological and inflammatory data as complementary evidence to strengthen the diagnosis. Thus, it is observed that laboratory parameters, while not primary differentiators on their own, play a critical role in enriching the overall diagnostic picture.

The observed divergences across the feature importance profiles of the retained base learners highlight the risks of relying on a single model. While clinically meaningful predictors such as radiological findings were consistently prioritized, other results reflected model-specific patterns. A notable example is the XGBoost model, which ranked fatigue as the second most important predictor despite its limited diagnostic value for differentiating pneumonia from bronchitis. This illustrates how individual learners may capture spurious correlations. Such findings reinforce the rationale of the DAPLEX framework: by aggregating importance profiles through a consensus mechanism, model-specific artifacts are mitigated, resulting in a more robust and clinically reliable importance profile.

To achieve this consensus, the Borda count method was employed to aggregate the individual model rankings. The top five predictors from this ensemble-based ranking are presented in Table 3.

The consensus ranking presented in Table 3 confirms that the ensemble’s diagnostic inference is informed by a clinically diverse and complementary set of predictors. Notably, the top-ranked features span multiple clinical domains, including a definitive radiological indicator, key physical examination findings (Rhonchus, Prolonged expiration), a core symptom (Fever), and an inflammatory biomarker (CRP). This multidimensional predictor profile demonstrates that the DAPLEX framework leverages integrated signals across categorical, symptomatic, and laboratory inputs, ensuring that its decisions are not dominated by any single domain but reflect a synthesized, evidence-based perspective.

Derived from Borda-based aggregation, the five consensus-driven features (the “why”) were integrated with the out-of-fold predicted probabilities from each base learner (the “what”) to construct a hybrid meta-input for the final decision layer. The final meta-learner, a Multilayer Perceptron (MLP), was chosen due to its capacity to model non-linear interactions and effectively fuse heterogeneous sources of information. To ensure both optimality and methodological consistency, hyperparameter tuning for the MLP was conducted using the same grid search strategy employed for the base learners. Details of the hyperparameter grid and the selected optimal settings are provided in Supplementary Table S5, ensuring full transparency and reproducibility.

##### Final Model Performance and Comparative Analysis

Before the diagnostic performance of the final, optimized DAPLEX framework was benchmarked against the individual base learners on the independent holdout test set, the stability of the Phase III meta-learner (MLP) was first evaluated through 5-fold cross-validation on the meta-training data. This crucial step was performed to ensure that the final integrated model was itself robust and not prone to overfitting. The results of this stability analysis are presented in Table 4.

Table 4 summarizes the cross-validation results of the final DAPLEX trained on the meta-training data, demonstrating both strong predictive capacity and high stability across folds. The model achieved a mean BA of 0.9377 ± 0.0108 with a minimal stability gap (Δ_BA_ = 0.0039), confirming that performance was consistent and generalizable. Sensitivity (0.9186 ± 0.0211; Δ = 0.0057) and specificity (0.9568 ± 0.0275; Δ = 0.0021) indicate that the DAPLEX effectively balanced FN and FP, while precision (0.9612 ± 0.0276; Δ = 0.0036) and F1-score (0.9389 ± 0.0116; Δ = 0.0048) further highlight its robust classification ability. The excellent ROC-AUC (0.9836 ± 0.0028; Δ = 0.0026) underscores strong discriminative power across thresholds, and the low Brier score (0.0489 ± 0.0094; Δ = 0.0065) confirms well-calibrated probability estimates.

When benchmarked against the individual base learners, the final DAPLEX model’s stability (Δ_BA_ = 0.0039) was on par with the most stable candidates like RF and SVM (RBF), and clearly superior to the only pruned model, KNN (Δ_BA_ = 0.0645). Collectively, these findings establish the final DAPLEX model as a highly consistent and reliable integrative framework, with intrinsic stability that minimizes the risk of overfitting and confirms the model’s generalizability, thereby providing a strong foundation for clinical prediction.

**Figure 8:**
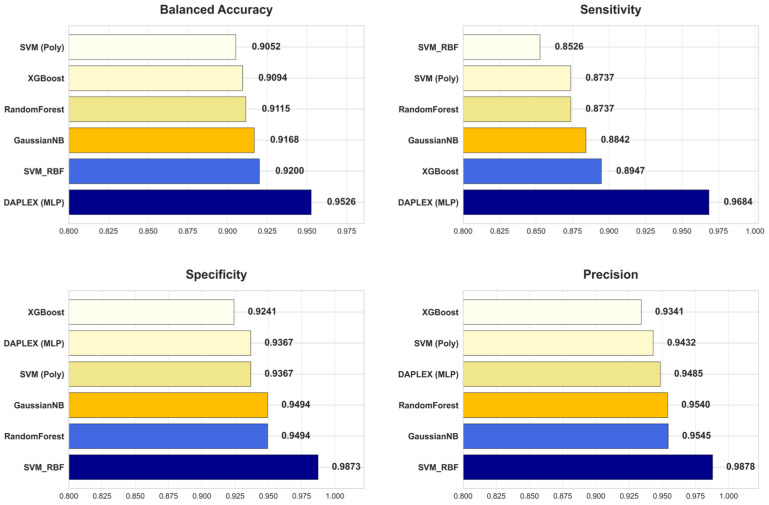

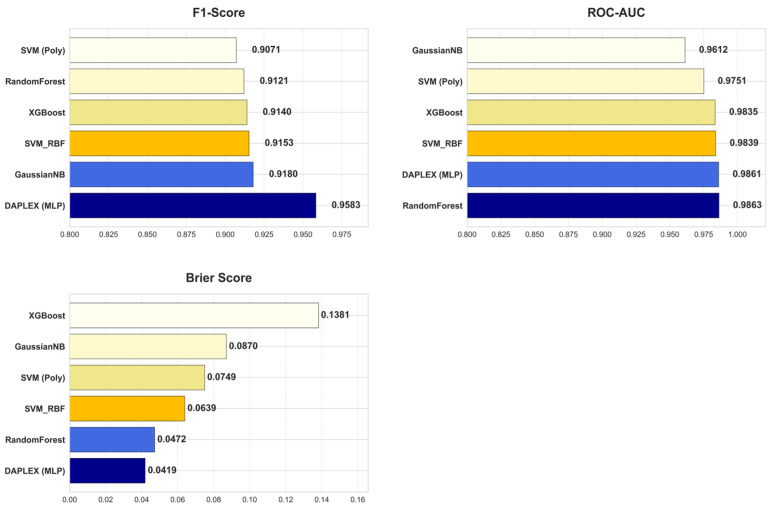
Comparative Diagnostic Performance of DAPLEX vs. Individual Base Learners Across Key Metrics.

The diagnostic performance of the final, optimized DAPLEX framework was benchmarked against the individual base learners on the independent holdout test set, using two core dimensions of model behavior: discriminative performance and calibration reliability. The comparative results are summarized in Figure 8, which provides a side-by-side evaluation across key metrics including BA, sensitivity, specificity, precision, F1-score, ROC-AUC, and Brier score.

DAPLEX, using MLP as the meta-learner, achieved the highest BA (0.9526) on the independent test set, clearly outperforming all individual base learners (range: 0.9052–0.9200). This substantial improvement indicates that the integrative framework effectively balanced sensitivity and specificity, minimizing both FN and FP. Beyond BA and sensitivity, DAPLEX also demonstrated consistent superiority in other complementary metrics. Its precision (0.9485) was comparable to high-performing tree-based models such as RF (0.9540) and GNB (0.9545), ensuring reliable positive predictions. The model’s F1-score (0.9583) exceeded all alternatives (range: 0.9071–0.9180), underscoring its ability to achieve a strong precision–recall balance. Furthermore, DAPLEX achieved one of the best ROC-AUC values (0.9861), on par with RF (0.9863) and slightly above XGBoost (0.9835), confirming its robust discriminative power across thresholds. Importantly, calibration analysis revealed that DAPLEX attained the lowest Brier score (0.0419), indicating that its predicted probabilities were the most reliable and well-calibrated among all evaluated models.

In quantitative terms, DAPLEX demonstrated significant gains over the retained base learners, a stable and methodologically diverse subset that emerged from the rigorous pruning procedure. Compared to the range of performance from the weakest to the strongest base learner, DAPLEX improved BA by 3.5–5.2%, enhanced the F1-score by 4.4–5.6%, and increased sensitivity by 8.2–13.6%. Most notably, in terms of calibration, it reduced the Brier score by 11.0% relative to the best-calibrated base learner (RF), and by a substantial 69.5% compared to the worst-calibrated model. This underscores its consistent and significant superiority in both discrimination and calibration. In terms of AUC, while RF recorded a slightly higher ROC-AUC (0.9863) compared to DAPLEX (0.9861), this marginal difference was clinically insignificant, indicating equivalent discriminative capacity for practical diagnostic use.

To ensure the clinical generalizability and fairness of the DAPLEX framework, a final post hoc analysis was conducted by stratifying the independent test set according to key demographic variables. Specifically, patients were grouped by sex (male vs. female) and by clinically relevant age categories defined according to the World Health Organization (WHO): infancy (0–11 months), early childhood (12–59 months), and school-aged childhood (≥60 months). The results, presented in Supplementary Table S6, demonstrated that the DAPLEX model maintained robust and consistent performance across all evaluated subgroups. BA remained high for both male (0.960) and female (0.941) patients, as well as across all age categories (range: 0.950–0.957). These findings provide strong evidence that the DAPLEX framework is not subject to systematic bias based on patient age or sex, thereby reinforcing its potential for reliable clinical application.

## 5. Discussion

This study introduced and validated DAPLEX, a structured, three-phase ensemble learning framework designed to differentiate pediatric pneumonia from acute bronchitis. The central finding is that DAPLEX achieved diagnostically superior and more reliable performance than any of its individually optimized base learners. These results underscore the value of a methodologically grounded ensemble strategy in complex clinical classification tasks, confirming that thoughtful model integration can significantly outperform stand-alone machine learning algorithms.

The findings align with and extend prior research on machine learning applications in pediatric respiratory diagnostics. While earlier studies demonstrated the utility of models such as KNN, RF, XGBoost, SVM and GNB for classifying LRTIs [20,83,84], they often lacked a systematic approach to ensemble construction. The present study addresses this methodological gap by illustrating that the architecture of ensemble development, particularly the deliberate selection of diverse and complementary models, is as critical as the performance of any single algorithm. This principle was quantitatively validated by the final DAPLEX model, which demonstrated intrinsic stability, minimizing the risk of overfitting and confirming its strong generalizability. Quantitatively, DAPLEX achieved notable improvements over individual base learners, with balanced accuracy increased by 3.5–5.2%, F1-score by 4.4–5.6%, and sensitivity by 8.2–13.6%. Calibration gains were particularly striking, with the Brier score reduced by 11.0% compared to the best-calibrated model and by 69.5% relative to the worst. Furthermore, the framework’s discriminative capacity was exceptional, achieving an ROC-AUC of approximately 0.99, which indicates near-optimal diagnostic performance.

This performance gain translates to clinically meaningful advantages. The final balanced accuracy of 95.3% signifies a high level of diagnostic power, robust enough to serve as a reliable aid in clinical decision-making. This translates to more equitable classification across both pneumonia and acute bronchitis, reducing the risk of two critical diagnostic errors: FN, which can delay essential treatment for pneumonia, and FP, which may lead to unnecessary antibiotic exposure. The quantitative improvements further underscore this. Compared to the best-performing individual base learner in each respective metric, the DAPLEX framework enhanced the F1-score by 4.4%, reflecting a more favorable balance between sensitivity and precision, a crucial factor in pediatric care where both overtreatment and undertreatment carry risks. Furthermore, the 11.0% reduction in the Brier score confirms that the DAPLEX produces highly calibrated probability estimates that align closely with actual clinical outcomes. This improved calibration enhances the model’s trustworthiness and supports more confident, evidence-based decision-making at the bedside

The success of the DAPLEX framework is attributable to its structured three-phase architecture, which systematically addresses key challenges in ensemble modeling through interdependent stages. Phase I established a rich predictive foundation by deploying a heterogeneous set of base learners from distinct algorithmic paradigms, thereby ensuring diversity as the prerequisite for later fusion. Phase II built directly on this foundation, acting as a critical quality-control gate where generalization stability was used to prune unreliable models such as KNN, thus safeguarding the integrity of inputs to the fusion stage. Finally, Phase III operationalized the core novelty of the framework: a complementarity-driven knowledge fusion that would not have been feasible without the heterogeneity created in Phase I or the reliability enforced in Phase II. By integrating not only the models’ predictive outputs but also their underlying feature-level insights, Phase III enabled the final meta-learner to operate on a richer and more trustworthy diagnostic representation than is available in conventional stacking approaches.

A key finding from the knowledge fusion phase was the consensus-based ranking of predictor importance, which aligns closely with established clinical pathophysiology. The top five predictors identified by the Borda count “Radiological findings”, “Rhonchus”, “Fever”, “Prolonged expiration”, and “CRP” span multiple clinical domains and reflect a holistic diagnostic signature. The high ranking of “Radiological findings” and “CRP” is consistent with literature that identifies them as strong indicators of the parenchymal inflammation characteristic of pneumonia [9,85]. Conversely, the prominence of auscultatory findings like “Rhonchus” and “Prolonged expiration” as key predictors aligns with their clinical role as signs of the airway obstruction that defines acute bronchitis [85]. The ability of the DAPLEX consensus mechanism to identify and prioritize this clinically validated set of multi-domain predictors underscores its potential to mimic expert diagnostic reasoning.

Despite its promising results, this study has several limitations that should be acknowledged. Methodologically, the DAPLEX framework was deliberately designed with modular flexibility, allowing it to adapt to heterogeneous data formats, preprocessing strategies, and ensemble fusion approaches. However, the present study evaluated this flexibility only within a retrospective, single-center, and binary diagnostic scenario. While this ensured high internal consistency from a standardized clinical workflow, it may limit the generalizability of the findings to other populations and healthcare systems. Moreover, the modular design was not tested under more complex settings such as multi-class diagnostic tasks or multimodal data integration (e.g., combining genomic or epidemiological information), which remain important directions for future work. Therefore, the highest priority for subsequent research is the prospective, multi-center validation of the DAPLEX framework to confirm its real-world clinical utility and to extend its evaluation to multi-class and multimodal diagnostic challenges. Finally, although the subgroup analysis indicated that DAPLEX maintained consistent performance across age and sex, the relatively small size of the test set limited the ability to draw more granular conclusions for other patient subgroups. Future studies on larger and more heterogeneous cohorts will be necessary to further validate the framework’s fairness and generalizability.

## 6. Conclusions

In conclusion, this study introduced and validated DAPLEX, a structured, complementarity-driven ensemble learning framework designed to address the diagnostic challenge of differentiating pediatric pneumonia from acute bronchitis. The results demonstrated that DAPLEX achieves both statistically significant and clinically meaningful improvements in diagnostic accuracy, calibration, and reliability compared to its constituent base learners. These gains are attributable to a systematic methodology that combines algorithmic diversity with a novel knowledge fusion strategy, integrating both prediction-level outputs and feature-level importance profiles. By offering a transparent, reproducible, and high-performing diagnostic pipeline, DAPLEX highlights the practical utility of multi-perspective modeling and reinforces the importance of structured ensemble approaches in complex clinical decision-making.

## Figures and Tables

**Table 1:** Comparative Cross-Validation Performance of Base Learner Models.

Metric	RF	XGBoost	SVM (RBF)	SVM (Poly)	KNN	GNB
BA	0.948 ± 0.016	0.936 ± 0.012	0.935 ± 0.021	0.895 ± 0.026	0.935 ± 0.016	0.925 ± 0.019
Sensitivity	0.905 ± 0.024	0.909 ± 0.031	0.878 ± 0.033	0.943 ± 0.019	0.896 ± 0.035	0.896 ± 0.038
Specificity	0.991 ± 0.011	0.964 ± 0.037	0.991 ± 0.011	0.847 ± 0.060	0.975 ± 0.011	0.955 ± 0.021
Precision	0.990 ± 0.013	0.966 ± 0.033	0.990 ± 0.014	0.877 ± 0.058	0.976 ± 0.013	0.961 ± 0.015
F1	0.943 ± 0.017	0.932 ± 0.010	0.929 ± 0.021	0.894 ± 0.026	0.931 ± 0.014	0.922 ± 0.020
ROC-AUC	0.985 ± 0.008	0.985 ± 0.006	0.983 ± 0.006	0.980 ± 0.007	0.973 ± 0.008	0.969 ± 0.010
Brier	0.056 ± 0.017	0.068 ± 0.011	0.071 ± 0.021	0.104 ± 0.027	0.068 ± 0.014	0.076 ± 0.019

**Table 2:** Stability Gap Analysis for Ensemble Pruning.

Base Learner	Stability Gap	Status
RF	Δ_BA_ = 0.0039	Retained
XGBoost	Δ_BA_ = 0.0140	Retained
SVM (RBF)	Δ_BA_ = 0.0019	Retained
SVM (Poly)	Δ_BA_ = 0.0032	Retained
KNN *	Δ_BA_ = 0.0645 *	Pruned *
GNB	Δ_BA_ = 0.0085	Retained

*. Values exceeding the stability gap threshold (Δ_BA_ = 0.05)

**Table 3:** Consensus Feature Importance Ranking via Borda Aggregation.

Rank	Feature Name	Feature Type	Borda Score
1	Radiological findings	Categorical	12
2	Rhonchus	Categorical	22
3	Fever	Categorical	24
4	Prolonged expiration	Categorical	29
5	CRP	Continuous	34

**Table 4:** Cross-Validation Stability of the Final DAPLEX Model.

Metric	Mean (CV)	Std Dev (CV)	Stability Gap (Δ)
BA	0.9377	0.0108	0.0039
Sensitivity	0.9186	0.0211	0.0057
Specificity	0.9568	0.0275	0.0021
Precision	0.9612	0.0276	0.0036
F1	0.9389	0.0116	0.0048
ROC-AUC	0.9836	0.0028	0.0026
Brier Score	0.0489	0.0094	0.0065

## Data Availability

Data are not publicly available due to privacy restrictions. The data that support the findings of this study are available from the corresponding author upon reasonable request.

## Supplementary Materials

The following supporting information can be downloaded at https://www.mdpi.com/article/10.3390/diagnostics15172258/s1, Table S1: Baseline Demographic and Clinical Characteristics of the Study Cohort, Stratified by Final Diagnosis; Table S2: Comparison of Baseline Characteristics Between the Training and Holdout Test Cohorts; Table S3: Hyperparameter Search Space and Optimal Configurations for Base Learners; Table S4: Comprehensive Stability Gap (Δ) Analysis Across All Performance Metrics; Table S5: Hyperparameter Search Space and Final Configuration for the MLP Meta-Learner; Table S6: Subgroup analysis of the final DAPLEX framework across demographic categories; Figure S1: Generalization Stability of Base Learners Across All Key Performance Metrics.
