# Supplementary material for: A Novel Knowledge Fusion Ensemble for Diagnostic Differentiation of Pediatric Pneumonia and Acute Bronchitis
Source: Diagnostics (Basel). 2025 Sep 6;15(17):2258. doi: 10.3390/diagnostics15172258 (PMC12427965; doi:10.3390/diagnostics15172258)

Table S1. Baseline Demographic and Clinical Characteristics of the Study Cohort, Stratified by Final Diagnosis.

Characteristic	Pneumonia (n=474)	Bronchitis (n=394)	Overall (n=868)	P-value
**Demographics**				
Age (months)	53.17 ± 2.44	35.90 ± 2.04	45.33 ± 1.65	<0.001*
Sex				0.159
Female	199 (42.0%)	146 (37.1%)	345 (39.7%)	
Male	275 (58.0%)	248 (62.9%)	523 (60.3%)	
**Laboratory Parameters**				
White blood cell count	11.93 ± 0.35	12.33 ± 0.27	12.11 ± 0.22	0.365
Neutrophil percentage	53.50 ± 0.99	52.74 ± 1.12	53.15 ± 0.74	0.614
Lymphocyte percentage	35.35 ± 0.89	36.90 ± 1.03	36.05 ± 0.67	0.252
C-reactive protein (CRP)	40.40 ± 3.07	14.23 ± 1.06	28.52 ± 1.80	<0.001*
Oxygen saturation (SpO_2)	94.50 ± 0.21	94.46 ± 0.20	94.48 ± 0.15	0.870
**Symptomatology**				
Fever				<0.001*
Yes	366 (77.2%)	150 (38.1%)	516 (59.4%)	
No	108 (22.8%)	244 (61.9%)	352 (40.6%)	
Cough				0.111
Yes	469 (98.9%)	394 (100.0%)	863 (99.4%)	
No	5 (1.1%)	0 (0.0%)	5 (0.6%)	
Respiratory distress				<0.001*
Yes	343 (72.4%)	352 (89.3%)	695 (80.1%)	
No	131 (27.6%)	42 (10.7%)	173 (19.9%)	
Chest pain				0.313
Yes	57 (12.0%)	38 (9.6%)	95 (10.9%)	
No	417 (88.0%)	356 (90.4%)	773 (89.1%)	
Fatigue				0.823
Yes	208 (43.9%)	169 (42.9%)	377 (43.4%)	
No	266 (56.1%)	225 (57.1%)	491 (56.6%)	
Feeding difficulty				0.308
Yes	269 (56.8%)	238 (60.4%)	507 (58.4%)	
No	205 (43.2%)	156 (39.6%)	361 (41.6%)	
Myalgia				0.038*
Yes	106 (22.4%)	65 (16.5%)	171 (19.7%)	
No	368 (77.6%)	329 (83.5%)	697 (80.3%)	
**Physical Examination**				
Weight-to-height ratio	0.95 ± 0.01	0.96 ± 0.01	0.95 ± 0.01	0.457
Respiratory rate	38.65 ± 0.60	43.66 ± 0.61	40.93 ± 0.43	<0.001*
Cyanosis				0.322
Yes	13 (2.7%)	6 (1.5%)	19 (2.2%)	
No	461 (97.3%)	388 (98.5%)	849 (97.8%)	

Tachypnea				<0.001*
Yes	329 (69.4%)	345 (87.6%)	674 (77.6%)	
No	145 (30.6%)	49 (12.4%)	194 (22.4%)	
Tachycardia				<0.001*
Yes	368 (77.6%)	362 (91.9%)	730 (84.1%)	
No	106 (22.4%)	32 (8.1%)	138 (15.9%)	
Nasal discharge				0.604
Yes	71 (15.0%)	65 (16.5%)	136 (15.7%)	
No	403 (85.0%)	329 (83.5%)	732 (84.3%)	
Sneezing				0.582
Yes	66 (13.9%)	61 (15.5%)	127 (14.6%)	
No	408 (86.1%)	333 (84.5%)	741 (85.4%)	
Nasal flaring				<0.001*
Yes	239 (50.4%)	286 (72.6%)	525 (60.5%)	
No	235 (49.6%)	108 (27.4%)	343 (39.5%)	
Intercostal retractions				<0.001*
Yes	263 (55.5%)	304 (77.2%)	567 (65.3%)	
No	211 (44.5%)	90 (22.8%)	301 (34.7%)	
Rhonchus				<0.001*
Yes	88 (18.6%)	389 (98.7%)	477 (55.0%)	
No	386 (81.4%)	5 (1.3%)	391 (45.0%)	
Crackles				<0.001*
Yes	433 (91.4%)	70 (17.8%)	503 (57.9%)	
No	41 (8.6%)	324 (82.2%)	365 (42.1%)	
Prolonged expiration				<0.001*
Yes	70 (14.8%)	381 (96.7%)	451 (52.0%)	
No	404 (85.2%)	13 (3.3%)	417 (48.0%)	
Hyperpnea				<0.001*
Yes	309 (65.2%)	338 (85.8%)	647 (74.5%)	
No	165 (34.8%)	56 (14.2%)	221 (25.5%)	
**Radiological Assessment**				
Radiological findings				<0.001*
Normal	8 (1.7%)	28 (7.1%)	36 (4.1%)	
Increased aeration and air bronchogram	60 (12.7%)	363 (92.1%)	423 (48.7%)	
Pneumonic infiltration	406 (85.7%)	3 (0.8%)	409 (47.1%)	

Table S2. Comparison of Baseline Characteristics Between the Training and Holdout Test Cohorts.

Characteristic	Train Set (N=694)	Test Set (N=174)	P-Value
**Age (months)**	45.27 ± 48.46	45.59 ± 49.09	0.938
**Weight-to-height ratio**	0.96 ± 0.18	0.95 ± 0.17	0.637
**Respiratory rate**	40.93 ± 12.95	40.90 ± 12.28	0.979
**White blood cell count**	12.22 ± 6.84	11.67 ± 5.46	0.325
**Neutrophil percentage**	52.81 ± 21.72	54.52 ± 22.68	0.358
**Lymphocyte percentage**	36.30 ± 19.70	35.08 ± 20.24	0.468
**C-reactive protein (CRP)**	27.20 ± 50.13	33.77 ± 62.81	0.144
**Oxygen saturation (SpO_2)**	94.51 ± 4.17	94.36 ± 4.90	0.666
**Sex**	Female: 271 (39.0%)<br>Male: 423 (61.0%)	Female: 74 (42.5%)<br>Male: 100 (57.5%)	0.452
**Fever**	Yes: 416 (59.9%)<br>No: 278 (40.1%)	Yes: 100 (57.5%)<br>No: 74 (42.5%)	0.612
**Cough**	Yes: 690 (99.4%)<br>No: 4 (0.6%)	Yes: 173 (99.4%)<br>No: 1 (0.6%)	1.000
**Respiratory distress**	Yes: 555 (80.0%)<br>No: 139 (20.0%)	Yes: 140 (80.5%)<br>No: 34 (19.5%)	0.970
**Chest pain**	Yes: 77 (11.1%)<br>No: 617 (88.9%)	Yes: 18 (10.3%)<br>No: 156 (89.7%)	0.883
**Fatigue**	Yes: 300 (43.2%)<br>No: 394 (56.8%)	Yes: 77 (44.3%)<br>No: 97 (55.7%)	0.874
**Feeding difficulty**	Yes: 394 (56.8%)<br>No: 300 (43.2%)	Yes: 113 (64.9%)<br>No: 61 (35.1%)	0.062
**Myalgia**	Yes: 139 (20.0%)<br>No: 555 (80.0%)	Yes: 32 (18.4%)<br>No: 142 (81.6%)	0.705
**Cyanosis**	Yes: 13 (1.9%)<br>No: 681 (98.1%)	Yes: 6 (3.4%)<br>No: 168 (96.6%)	0.327
**Tachypnea**	Yes: 542 (78.1%)<br>No: 152 (21.9%)	Yes: 132 (75.9%)<br>No: 42 (24.1%)	0.595
**Tachycardia**	Yes: 588 (84.7%)<br>No: 106 (15.3%)	Yes: 142 (81.6%)<br>No: 32 (18.4%)	0.374
**Nasal discharge**	Yes: 109 (15.7%)<br>No: 585 (84.3%)	Yes: 27 (15.5%)<br>No: 147 (84.5%)	1.000
**Sneezing**	Yes: 102 (14.7%)<br>No: 592 (85.3%)	Yes: 25 (14.4%)<br>No: 149 (85.6%)	1.000
**Nasal flaring**	Yes: 416 (59.9%)<br>No: 278 (40.1%)	Yes: 109 (62.6%)<br>No: 65 (37.4%)	0.572
**Intercostal retractions**	Yes: 453 (65.3%)<br>No: 241 (34.7%)	Yes: 114 (65.5%)<br>No: 60 (34.5%)	1.000
**Rhonchus**	Yes: 380 (54.8%)<br>No: 314 (45.2%)	Yes: 97 (55.7%)<br>No: 77 (44.3%)	0.881
**Crackles**	Yes: 407 (58.6%)<br>No: 287 (41.4%)	Yes: 96 (55.2%)<br>No: 78 (44.8%)	0.457

**Prolonged expiration**	Yes: 359 (51.7%)<br>No: 335 (48.3%)	Yes: 92 (52.9%)<br>No: 82 (47.1%)	0.853
**Hyperpnea**	Yes: 519 (74.8%)<br>No: 175 (25.2%)	Yes: 128 (73.6%)<br>No: 46 (26.4%)	0.816
**Radiological findings**	Normal: 29 (4.2%)<br>Increased aeration: 335 (48.3%)<br>Pneumonic infiltration: 330 (47.6%)	Normal: 7 (4.0%)<br>Increased aeration: 88 (50.6%)<br>Pneumonic infiltration: 79 (45.4%)	0.862

Table S3. Hyperparameter Search Space and Optimal Configurations for Base Learners.

Model	Hyperparameter Search Space	Optimal Configuration
**Random Forest**	n_estimators: [20, 30, 40, 50]; max_depth: [2, 3, 4]; min_samples_leaf: [20, 25, 30, 35]; min_samples_split: [10, 15, 20]; max_features: [0.1, 0.2, 0.3, 0.4]; bootstrap: [True]; criterion: ['gini', 'entropy']	{'bootstrap': True, 'criterion': 'entropy', 'max_depth': 3, 'max_features': 0.4, 'min_samples_leaf': 20, 'min_samples_split': 10, 'n_estimators': 40}
**XGBoost**	n_estimators: [10, 20, 30, 40, 50]; learning_rate: [0.001, 0.01]; max_depth: [2, 3, 5]; subsample: [0.4, 0.5, 0.6]; colsample_bytree: [0.3, 0.5, 0.6]; gamma: [0, 1]; reg_alpha: [1, 5]; reg_lambda: [3, 5, 10]	{'colsample_bytree': 0.3, 'gamma': 0, 'learning_rate': 0.01, 'max_depth': 5, 'n_estimators': 50, 'reg_alpha': 1, 'reg_lambda': 3, 'subsample': 0.5}
**SVM (RBF Kernel)**	C: [0.0001, 0.001, 0.01, 0.1]; kernel: ['rbf']; gamma: [0.05, 0.01, 0.001]; probability: [True]	{'C': 0.1, 'gamma': 0.01, 'kernel': 'rbf', 'probability': True}
**SVM (Polynomial Kernel)**	C: [0.001, 0.01]; kernel: ['poly']; degree: [2, 3, 4]; gamma: ['scale', 'auto']; probability: [True]	{'C': 0.01, 'degree': 3, 'gamma': 'scale', 'kernel': 'poly', 'probability': True}
**K-Nearest Neighbors**	n_neighbors: [3, 5, 7, 9, 11]; weights: ['uniform', 'distance']; p: [1, 2]	{'n_neighbors': 5, 'p': 1, 'weights': 'distance'}
**Gaussian Naive Bayes**	var_smoothing: [1e-6, 1e-5, 1e-4]	{'var_smoothing': 1e-06}

Table S4. Comprehensive Stability Gap (Δ) Analysis Across All Performance Metrics.

Model	$\Delta_{sensitivity}$	$\Delta_{specificity}$	$\Delta_{precision}$	Δ_{f1}	Δ_{roc_auc}	Δ_{brier}
**RandomForest**	0,0118	0,0040	0,0017	0,0055	0,0069	0,0051
**XGBoost**	0,0196	0,0084	0,0110	0,0169	0,0056	0,0166
**KNN**	0,1043	0,0247	0,0238	0,0686	0,0274	0,0678
**SVM_RBF**	0,0030	0,0007	0,0011	0,0011	0,0017	0,0018
**GaussianNB**	0,0026	0,0144	0,0115	0,0078	0,0051	0,0069
**SVM_SimplePoly**	0,0021	0,0084	0,0128	0,0065	0,0025	0,0065

Table S5. Hyperparameter Search Space and Final Configuration for the MLP Meta-Learner.

**Hyperparameter**	**Hyperparameter Search Space**	**Optimal Configuration**
**Activation Function**	['tanh']	tanh
**Alpha (L2 penalty)**	[0.0005, 0.001, 0.002, 0.005]	0.005
**Hidden Layer Sizes**	[(50,), (80,), (100,), (100, 50), (120,), (80, 40), (120, 60)]	(50,)
**Learning Rate (init)**	[0.005, 0.01, 0.015, 0.02]	0.02
**Batch Size**	[4, 8, 12, 16, 32]	16
**Max Iterations**	Fixed at 5000	5000
**Early Stopping**	Enabled (n_iter_no_change=30)	Enabled
**Dropout**	Not available in scikit-learn MLPClassifier	Not used
**Batch Normalization**	Not available in scikit-learn MLPClassifier	Not used
**Optimizer (Solver)**	{'adam'}	adam
**Weight Initialization**	Handled internally by scikit-learn (activation-specific, Xavier/Glorot scheme)	Default (scikit-learn)

Table S6. Subgroup analysis of the final DAPLEX framework across demographic categories.

Subgroup	Category	N (Patients)	Balanced Accuracy	F1-Score	ROC-AUC	Brier Score
**Overall**	All Patients	174	0.9526	0.9583	0.9861	0.0419
**Sex**	Female	74	0.9412	0.9535	0.9859	0.0492
	Male	100	0.9599	0.9623	0.9864	0.0365
**Age Group**	<12 months	52	0.9500	0.9362	0.9955	0.0447
	12–59 months	70	0.9571	0.9565	0.9788	0.0436
	≥60 months	52	0.9511	0.9737	0.9887	0.0369

Figure S1. Generalization Stability of Base Learners Across All Key Performance Metrics.

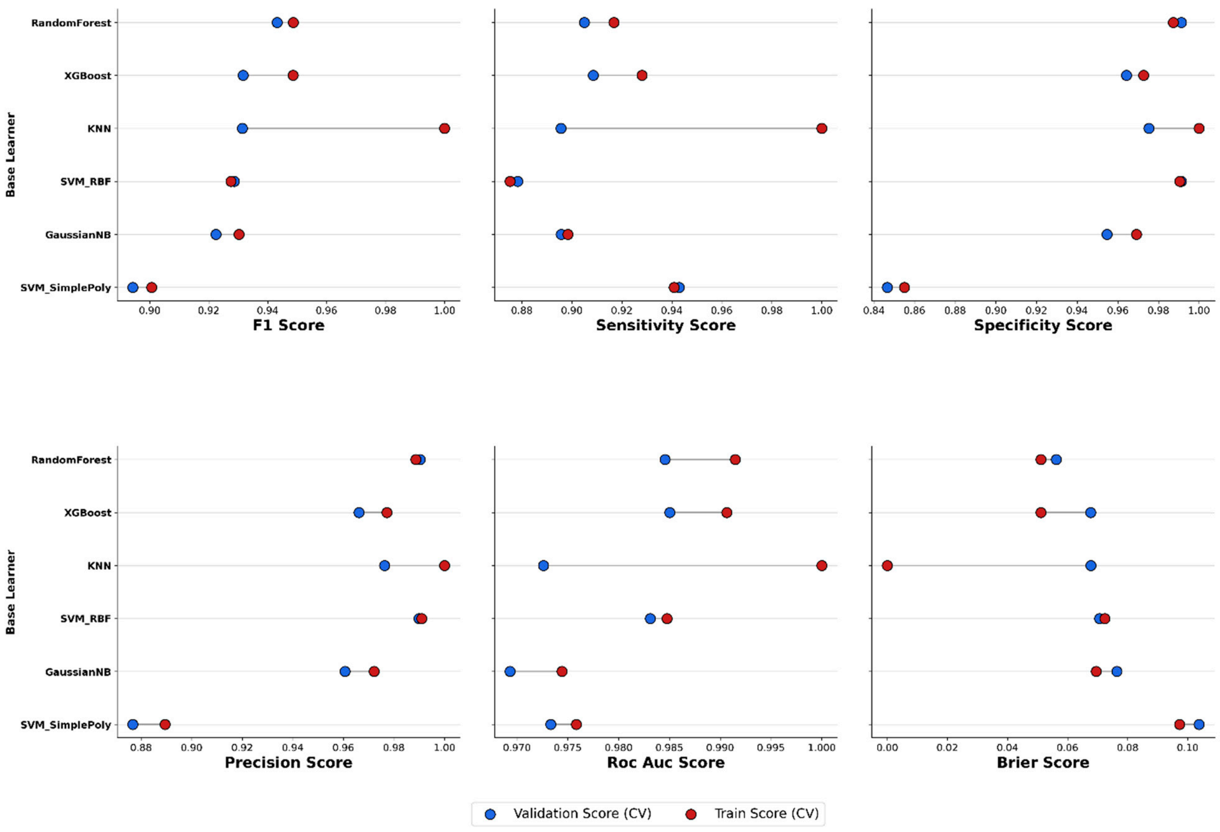

Supplement: Supplementary file 1 [file diagnostics-15-02258-s001.zip › diagnostics-3805416-supplementary.pdf]
